# Optimizing Telehealth: Leveraging Key Performance Indicators for Enhanced TeleHealth and Digital Healthcare Outcomes (Telemechron Study)

**DOI:** 10.3390/healthcare12131319

**Published:** 2024-07-01

**Authors:** Sandra Morelli, Carla Daniele, Giuseppe D’Avenio, Mauro Grigioni, Daniele Giansanti

**Affiliations:** Centro Nazionale per le Tecnologie Innovative in Sanità Pubblica, Istituto Superiore di Sanità, Via Regina Elena 299, 00161 Roma, Italy; sandra.morelli@iss.it (S.M.); carla.daniele@iss.it (C.D.); giuseppe.davenio@iss.it (G.D.); mauro.grigioni@guest.iss.it (M.G.)

**Keywords:** telemedicine, digital healthcare, telehealth, key performance indicator, quality

## Abstract

Over the past decade, the use of telehealth has garnered increasing attention. The focus on quality aspects has seen significant growth in tandem with the telehealth expansion. Having useful indicators in this area is becoming increasingly strategic for fully integrating the technology into the health domain. These indicators can help monitor and evaluate the quality of telehealth services, guiding improvements and ensuring that these digital solutions meet the necessary standards for effective healthcare delivery. The purpose of this study is to analyze Key performance indicators (KPIs) in telehealth within institutional websites and the scientific dissemination world by means of a narrative review. A narrative review was proposed with these two specific points of view based on a standardized checklist and a quality control procedure for including scientific papers in the analysis. Results from scientific studies emphasize KPIs such as patient outcomes, operational efficiency, technical reliability, and cost-effectiveness. These include measures like improvements in condition management, patient satisfaction, consultation numbers, waiting times, and cost savings. Institutional documents from entities like the WHO also show diverse perspectives, focusing on equitable access, clinical excellence, patient prioritization, response times, and patient and staff satisfaction. The findings suggest that adopting a comprehensive set of KPIs and continuously monitoring and evaluating telehealth services can enhance their effectiveness, efficiency, and equity, ultimately improving healthcare outcomes and accessibility.

## 1. Introduction

The use of telehealth has gained significant traction over the past decade, with a notable surge in attention during the COVID-19 pandemic [1]. The pandemic accelerated the adoption of telehealth by overcoming longstanding barriers [2,3], positioning it as a crucial tool for remote care and safeguarding vulnerable populations [4,5]. This period underscored telehealth’s potential to revolutionize healthcare delivery, ensuring continuous care while minimizing infection risks and optimizing resources [6,7]. Innovations and lessons from this transformative period continue to influence the future of digital healthcare.

**Figure 1:**
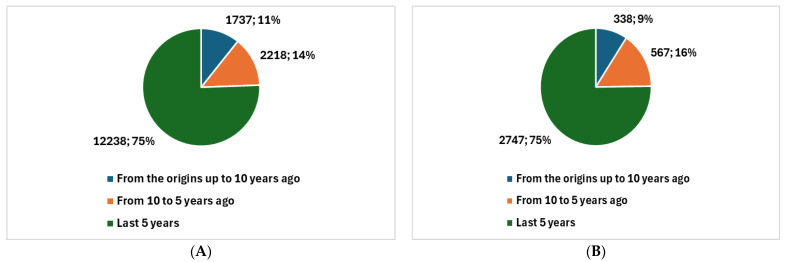
(**A**) Trends of studies in telehealth over the years. (**B**) Trends of studies in telehealth with focus also on quality over the years.

When we examine the trends in telehealth on PubMed (see Box S1-position 1 in Supplementary Material) we observe that as of today, since 1978, a total of 16,193 [8] studies have been generated. Of these, 14,456 were produced in the last decade, while 12,238 were produced in the last five years, marked by the pandemic (Figure 1A).

An important aspect of these systems is the quality of service related to their use. Quality considerations encompass a multitude of domains, including the interactions between the patient and the healthcare provider connected virtually to the system. Ensuring high-quality service in telehealth involves addressing factors such as the reliability and security of the technology, the effectiveness of the communication be-tween patients and providers, and the overall user experience for both parties.

The focus on quality aspects has seen significant growth in tandem with the tele-health expansion. The earliest visible studies in this domain on PubMed (see Box S1-position 2 in Supplementary Material) date back to 1989, with a total of 3652 studies produced to date [9]. Among these, 3314 were conducted in the past decade, while 2747 were conducted in the last five years, coinciding with the pandemic (Figure 1B). Comparing Figure 1A to Figure 1B, it is evident that both telehealth studies and those specifically focusing on quality in telehealth have collectively accounted for 75% of all historical production in the last five years.

Having useful indicators in this area is becoming increasingly strategic for fully integrating technology into the health domain. These indicators can help monitor and evaluate the quality of telehealth services, guiding improvements and ensuring that these digital solutions meet the necessary standards for effective healthcare delivery. These indicators can measure various aspects such as patient satisfaction, response times, system uptime, and clinical outcomes, providing a comprehensive view of the service quality.

By leveraging these indicators, healthcare organizations can better understand the impact of telehealth on patient care and make informed decisions to enhance service delivery. This strategic approach ensures that telehealth technology is effectively embedded into healthcare systems, optimizing its benefits and improving overall healthcare outcomes.

However, while studies dedicated to quality are numerous, those focusing on indicators are scarce. A search on PubMed (see Box S1-position 3 in Supplementary Material) reveals that since 2012 [10], only 17 studies have been published, with 14 of them appearing in the last five years marked by the pandemic. Among these studies, only two are reviews [11,12], with one being more recent and related to the pandemic [11].

The first review [12] delves into the relationship between telehealth and patient satisfaction, homing in on key indicators like improved outcomes, preferred modality, ease of use, cost-effectiveness, communication quality, and reduced travel time. By analysing 44 pertinent articles from a pool of 2193 through systematic review, it’s evident that these indicators play a pivotal role, collectively representing 61% of mentions. Understanding and addressing these indicators can significantly enhance telehealth interventions, ensuring better patient satisfaction and overall care quality.

The second review [11] in the pandemic era examines how the COVID-19 pandemic affected hospital cardiac care using performance indicators. From a scoping review of 6277 articles, 94 were included, revealing 1637 indicators. Most indicators showed decreases in admissions, delays in patient presentation, and worsened clinical conditions. Diagnostic and treatment procedures decreased, while telehealth utilization increased. Length of stay decreased, and acute coronary syndrome treatment times increased. Outpatient activity declined, and mortality rates rose. These findings underscore the pandemic’s widespread impact on cardiac care, aiding future planning efforts.

The two studies [11,12] highlight that indicators, acting as critical sensors for the quality system, are essential for effectively monitoring and evaluating telehealth applications. These indicators provide valuable insights into the performance and impact of telehealth services, ensuring that they meet the required standards and deliver optimal patient outcomes. A comprehensive approach to these indicators should cover multiple domains, including clinical effectiveness, patient satisfaction, operational efficiency, and economic impact.

Recently, there has been significant discussion around the importance of Key Performance Indicators (KPIs) in assessing the performance of various systems, including telehealth. KPIs are specific, measurable metrics that help healthcare providers track and evaluate the success of their telehealth programs. By focusing on these indicators, healthcare organizations can identify areas for improvement, optimize resource allocation, and enhance overall service quality. Key Performance Indicators (KPIs) are “the critical (key) quantifiable indicators of progress toward an intended result. KPIs provide a focus for strategic and operational improvement, create an analytical basis for decision making and help focus attention on what matters most” (literal citation) [13].

Managing with KPIs involves setting targets and tracking progress, using leading indicators to predict success and lagging indicators to measure outcomes [13]. Good KPIs [13,14] provide objective evidence of progress, measure intended metrics, offer performance comparisons over time, and track various aspects like efficiency, quality, and resource utilization. By balancing leading and lagging indicators, KPIs ensure effective monitoring and improvement of telehealth applications.

The use of Key Performance Indicators (KPIs) in healthcare is increasingly recognized and documented [15,16,17], highlighting their pivotal role in monitoring and enhancing healthcare outcomes. KPIs provide critical insights into healthcare system performance, facilitating progress tracking, identifying areas for improvement, and supporting informed decision-making. In telehealth, which has rapidly adopted digital technologies for remote care [18,19], integrating KPIs represents a significant advancement. These metrics systematically evaluate telehealth initiatives, assessing patient outcomes, care access, efficiency, and cost-effectiveness. However, deploying KPIs in telehealth poses unique challenges such as data accuracy, privacy concerns, and equitable service access, especially for underserved populations. Addressing these challenges requires collaborative efforts across healthcare stakeholders, policymakers, and technology developers to standardize metrics, improve data security, and expand telehealth infrastructure. Effective use of KPIs is crucial for optimizing telehealth’s impact on healthcare delivery and patient outcomes as it continues to evolve.

### Purpose

The purpose of this study is to analyze KPIs in telehealth within institutional websites and the scientific dissemination world by means of a narrative review. By examining KPIs specific to telehealth, this research aims to understand how healthcare organizations and scientific communities utilize these metrics to monitor, evaluate, and optimize telehealth services. The study seeks to identify common KPIs employed in telehealth initiatives, assess their effectiveness in measuring performance and outcomes, and explore any challenges or trends associated with their implementation. Through this analysis, the study aims to provide insights that can inform best practices and guide future developments in telehealth evaluation and management.

Some of the questions that the narrative review could address include:

- What are the key challenges and opportunities associated with implementing telehealth KPIs?
- How do telehealth KPIs contribute to improving healthcare delivery and patient outcomes?
- What are the most commonly used telehealth KPIs, and how do they vary across different healthcare settings?
- How do healthcare organizations and stakeholders utilize telehealth KPIs for performance measurement and quality improvement?

## 2. Materials and Methods

The two different polarities of the narrative review serve to address distinct aspects of the research:

The first polarity, based on a search of publication databases, aims to gather peer-reviewed literature and research studies related to telehealth Key Performance Indicators (KPIs). This involves analyzing existing studies to identify trends, challenges, and effectiveness of telehealth KPIs in healthcare settings.

The second polarity involves investigating how healthcare organizations use and report on telehealth KPIs by examining their institutional websites. This process includes reviewing various reports, guidelines, and documentation that these organizations publish. Through this examination, we aim to understand the specific KPIs they use to measure telehealth performance and outcomes. By assessing these documents, we can discern the recommended practices for implementing and evaluating telehealth services. Another critical aspect is evaluating the transparency and comprehensiveness of the KPI reporting on these websites. This involves determining how detailed and transparent the organizations are in sharing their telehealth performance metrics, and whether the reports cover a wide range of KPIs in a thorough manner. The goals of this exploration are to understand the various approaches healthcare organizations take to measure telehealth performance, identify best practices and benchmarks in KPI reporting, and highlight any gaps in transparency or comprehensiveness. Through this investigation, we can gain valuable insights into the effectiveness and reporting practices of telehealth programs in different healthcare settings.

The research, therefore, does not necessarily focus on best of the bunch institution in the field but rather on those that offer easy access to information about their KPIs directly from their websites. The goal is to find data that is readily available without the need for deferred interactions, such as contacting specific representatives or requesting secondary level access.

This approach facilitates the collection of immediate and accessible information on how healthcare organizations measure and report their performance in telemedicine and telehealth. This methodology allows for a practical and timely overview of the KPI approaches used by various organizations, without the need to wait for additional responses or permissions. It is evident that adopting an approach that involves deferred interactions, such as reaching out to specific representatives or requesting secondary access levels, has the potential to greatly broaden the scope of institutional sources considered, encompassing diverse sources from various countries around the world. However, this broader scope comes with a potential drawback: it can lead to a significant increase in the time required to conduct the study. The need to navigate multiple channels, coordinate with various representatives, and await responses could result in delays that render the study impractical or less efficient. Balancing thoroughness with efficiency becomes crucial in ensuring that the research process remains manageable and insightful.

The overview of scientific literature used a standardized checklist designed for the narrative category of reviews (ANDJ Narrative Checklist. Available online: [20]. Given the specificity of investigation. The search was based on targeted searches on Pubmed, Scopus, and Google scholar. 

The components of the overview were obtained by means of the combination of two groups of keywords also combined with AND/OR Boolean logic of search:

- Group 1: General Telemedicine and Telehealth
  Telemedicine
  Telehealth
  Remote Healthcare
  Virtual Care
  Health Technology
  Healthcare Delivery
  Telemedicine Assessment
  Telehealth Evaluation
- Group 2: Specific Metrics and Performance Indicators
  Key Performance Indicators (KPIs)
  Performance Metrics
  Healthcare Metrics
  Quality Metrics
  Outcome Measures
  Evaluation Criteria

These groups can help focus searches on either the broad context of telehealth and telemedicine or the specific performance and quality metrics used to evaluate these services.

Studies from journals and/or conferences had to be peer-reviewed to be included in the process of preselection below described.

The search was performed both in the [Title/abstract] and in the full text.

The choice of the component elements of this overview was made taking into account 5 parameters (N1–N5) evaluated with a score from 1 = minimum to 5 = maximum and one parameter (N6) with a binary assessment (Yes/No). These parameters have been identified into:

N1. Is the rationale for the study in the introduction clear?
N2. Is the design of the work appropriate?
N3. Are the methods described clearly?
N4 Are the results presented clearly?
N5. Are the conclusions based and justified by results?
N6. Did the authors disclosed all the conflict of interests?

All the selected studies had to have the parameter N6 with “Yes” and the parameters N1–N5 with a score > 3.

## 3. Results

The results are organized into two sections, corresponding to the two focal points outlined in the overview. The first section (Section 3.1) presents the outcomes of international scientific studies. The second section (Section 3.2) details the findings from an extensive survey of national and international websites to determine the stance of governmental and supranational organizations.

### 3.1. Outcome from the Overviewed Studies

The defined procedure led to the identification of 18 studies [21,22,23,24,25,26,27,28,29,30,31,32,33,34,35,36,37,38]. The section is divided into two paragraphs. The first paragraph (Section 3.1.1) presents the emerging common message, a categorization based on the evidence, and the related focus on KPIs, accompanied by detailed tables. The second paragraph (Section 3.1.2) provides an analytical summary.

#### 3.1.1. Common Message, Categorization, and Focus on the KPIs 

These studies underscore the transformative potential of telehealth in healthcare delivery, highlighted by the strategic integration of KPIs) across various initiatives. Research efforts, such as [21], emphasize the importance of standardized methodologies in developing KPIs for digital health interventions. The involvement of stakeholders is crucial for creating comprehensive evaluation frameworks.

Similarly, investigations like [22,23] explore telehealth’s impact on healthcare systems, demonstrating how KPIs serve as benchmarks for structural and procedural improvements. Initiatives dedicated to define models of telehealth [32] and shared telehealth service centers [37] illustrate the strategic use of KPIs to promote the adoption of telehealth, ensuring both effectiveness and efficiency.

In clinical contexts, telehealth is shown to be transformative, particularly in diabetic retinopathy screening [38], where KPIs are leveraged to improve screening rates and patient outcomes, positioning telehealth as a viable solution to healthcare challenges. Additionally, studies like [29,30] highlight the critical role of KPIs in assessing the usability, technical performance, and overall effectiveness of telehealth frameworks and models.

The impact of telehealth extends beyond clinical settings, as demonstrated by [31], which examines its long-term effects on emergency medical services (EMS) and the importance of KPIs in evaluating structural and operational aspects. Moreover, research such as [34] emphasizes the need for enhanced reporting on the implementation and planning of telehealth services, facilitating learning and sustainable service development.

Collectively, these findings from a diverse array of studies [21,22,23,24,25,26,27,28,29,30,31,32,33,34,35,36,37,38] underscore the pivotal role of KPIs in driving the success of telehealth initiatives, guiding decision-making, and ultimately advancing the quality and reach of healthcare services.

Table 1 reports a categorization of the studies. This categorization highlights the diverse focus areas and KPIs addressed across the studies, ranging from the development of KPIs for specific healthcare interventions to the evaluation of telehealth outcomes and system implementation. 

Table 2 reports a brief overview of each study’s key findings and emphasizes the significance of Key KPIs in evaluating various aspects of healthcare delivery in telehealth and technology integration.

#### 3.1.2. Analytical Summary

In this paragraph, an analytical summary is provided focusing on the topic of the overview, specifically highlighting the role of KPIs in telehealth. It delves into how these key performance indicators are essential for monitoring and enhancing the effectiveness of remote healthcare services. By evaluating aspects such as response times, problem resolution rates, appointment adherence, consultation durations, patient satisfaction, and other relevant metrics, KPIs enable healthcare providers to identify areas for improvement, streamline processes, and ensure high-quality, patient-centered telehealth services. Additionally, KPIs help in assessing the cost-effectiveness of telehealth programs, measuring clinical outcomes, ensuring regulatory compliance, facilitating data-driven decision-making to continuously enhance service delivery and other opportunities of the telehealth integration in the health domain [21,22,23,24,25,26,27,28,29,30,31,32,33,34,35,36,37,38].

Brenner et al. [21] remarks that digital health interventions (DHIs) provide innovative methods for delivering healthcare. KPIs are essential for evaluating, measuring, and improving healthcare quality and service performance. This scoping review aimed to identify current knowledge and evidence on developing KPIs for DHIs. A literature search across ten databases, including AMED, CINAHL, MEDLINE, and IEEE Xplore, identified five relevant references. These included two original research studies on specific DHIs and two overviews of methods for developing DHIs. All studies emphasized the importance of stakeholder involvement in KPI development, using various methodologies such as elicitation frameworks, factorial surveys, and Delphi studies. The review highlighted a significant gap in evidence-based knowledge on KPI development for DHIs. Despite the critical role of stakeholder involvement, there is a lack of standardized methodologies. Further research is needed to establish common methods for KPI development to improve comparability and advance the field.

The study proposed by Duong et al. [22] highlights that Teledermatology has emerged as a potential solution to enhance access and expedite skin cancer management, yet its impact on non-melanoma skin cancer (NMSC) care pathways remains unexplored. This study aimed to assess the effectiveness of teledermatology (TD) compared to conventional care pathways for NMSC management, focusing on KPIs. Conducted over two periods in a dermatology department, the study compared patient characteristics, tumor types, and processes between conventional care and post-TD diagnosis periods. Time was utilized as a crucial KPI to evaluate the efficiency of both pathways. Results revealed that despite TD-managed patients being significantly older, their total time spent in the process was not shorter compared to those managed conventionally. This unexpected finding underscores the importance of utilizing KPIs, like time, to comprehensively assess the impact of healthcare innovations, such as TD integration, on patient care pathways.

Another study proposed by Caffery et al. [23] investigated telehealth substitution rates at Princess Alexandra Hospital (PAH) in Brisbane, Australia, focusing on KPIs such as telehealth-eligible and overall telehealth substitution rates. The study highlighted the disparity between these rates, with the telehealth-eligible rate substantially higher due to geographical constraints. Notably, KPIs like telehealth activity as a reportable metric significantly influenced substitution rates, indicating the importance of KPIs in assessing the effectiveness of telehealth integration.

Clinical pharmacy quality indicators as highlighted by Canning et al. [24] often lack uniformity and fail to measure outcomes directly. This study aimed to establish a consensus on pharmaceutical care bundles and patient outcome measures across an entire state health service, focusing on KPIs for clinical pharmacy services. Using a modified Delphi approach with state Directors of Pharmacy, participants rated the relevance and measurability of 32 inpatient clinical pharmacy quality indicators and outcome measures on a Likert scale. Based on the results, pharmaceutical care bundles comprising multiple clinical pharmacy activities were formed, and their relevance and measurability were assessed. Results showed that consensus was reached on clinical pharmacy quality indicators, with the top indicator being accurate documentation of medicines upon admission. Out of nine proposed pharmaceutical care bundles, only one reached consensus, along with sixteen outcome measures, including hospital-acquired complications and unplanned readmissions. The next step is to assess the delivery of the agreed pharmaceutical care bundle and its impact on patient outcomes.

In another study performed by Garbelli et al. [25], the focus was on evaluating the relationship between the implementation of Fresenius Medical Care medical patient review CQI (MPR-CQI) and patients’ survival in end-stage kidney disease. The study included incident adult patients on chronic dialysis registered in the Europe, Middle East, and Africa region between 2011 and 2018. Results showed an increase in KPI target achievements after MPR-CQI policy implementation, with mediation analysis demonstrating a significant reduction in mortality due to an indirect effect of MPR-CQI implementation through improvement in KPI target achievement. The study suggests that standardized clinical practice and structured MPR may improve patients’ survival through enhancement of medical KPIs.

In [26] Jackson et al. the focus was on quantifying the use of evaluation criteria in telehealth and identifying current trends in metric adoption. The study aimed to determine the frequency of actual performance metric reporting in telehealth evaluation, emphasizing KPIs for clinical outcomes, satisfaction, patient quality, and cost measures. Using an automated literature search, telehealth studies reporting quantitative performance metrics were identified. Studies were categorized by telehealth class (store-and-forward, real-time consultation, telecare) and study stage (pilot vs. routine use). Evaluation metric reporting was analyzed across different telehealth classes and study stages. Results showed variations in metric reporting among telehealth classes and study stages, with different evaluation strategies observed. Clinical outcomes and patient satisfaction were frequently reported, but reporting of other performance metrics was rare.

In the study proposed by Baughman et al. [27], the focus was on comparing the quality of care between patients exposed to telehealth and those with only in-person office-based care. Quality measures based on specific KPIs, including Health Care Effectiveness Data and Information Set (HEDIS) performance measures, were compared between the two groups across outpatient care sites in Pennsylvania and Maryland. Results indicated that patients with telehealth exposure had better performance in testing-based and counseling-based measures compared to patients with only in-person office visits. Telehealth exposure was associated with significant improvements in various quality measures related to testing and counseling. The study suggested a largely favorable association between telehealth exposure and the quality of primary care, particularly in chronic disease management and preventive care. 

In Avanesova et al. [28], the focus was on analyzing research performance, international collaboration, corporate contribution, country-level economic factors, and legislative frameworks associated with the worldwide implementation of telehealth programs. Telehealth scholarly output and its association with various factors were analyzed using data from scholarly databases and the World Health Organization Global Observatory for eHealth. Results showed that research performance, training of medical students and healthcare professionals, collaboration with the technology industry, and legislative frameworks were associated with the implementation of telehealth programs. The study emphasized the importance of consistent collection and routine analysis, based also on specific KPIs, of patient outcomes after telehealth interventions and highlighted the need for international legislation to support telehealth capacity building, reimbursement policy, and secure data sharing policies.

In the study proposed by Berlet et al. [29], the focus was on the development and clinical evaluation of a 5G usability test framework for preclinical diagnostics using mobile ultrasound and 5G network technology. The study aimed to assess the usability and technical performance of 5G-enabled bidirectional data transmission between an ambulance car and a remote hospital site. The study evaluated various KPIs related to telemedical and clinical application properties, including ultrasound image quality, transmission latency, and data throughput. Results showed positive ratings for the usability and clinical applicability of the ultrasound probe, with bidirectional data transmission successfully established through the 5G network. Technical evaluations revealed an average end-to-end round trip latency of 10 milliseconds and measured throughput for ultrasound image traffic and video streaming. The study also highlighted the importance of core slicing for optimizing quality and latency in data transmission.

In Kidholm et al. [30] the focus was on assessing the face validity of the Model for Assessment of Telehealth (MAST), which serves as a framework for evaluating the effectiveness of telehealth applications. The importance of MAST domains and topics in relation to the KPIs have been highlighted in the study. The study aimed to determine the importance of different domains and topics in MAST as perceived by healthcare decision-makers and experts in telehealth. Using a modified Delphi process involving a workshop with healthcare decision-makers, the study assessed the importance of MAST domains and topics on a Likert scale. Results confirmed the face validity of all MAST domains, with more than 80% of respondents rating them as moderately or highly important. While the study supported the validity of MAST, suggestions for supplements and improvements regarding study design and data collection were noted, emphasizing the need for larger studies to confirm the results.

Schröder et al. [31] delved into the longitudinal effects of integrating a tele-EMS system on both the framework and operational facets of emergency medical services (EMS) in Aachen city from 2015 to 2021. With a keen focus on KPIs, the study aimed to gauge the efficacy of the tele-EMS implementation by scrutinizing structural and procedural metrics. Structurally, the study observed a notable surge in the total volume of EMS missions over the seven-year period, accompanied by a significant rise in tele-EMS consultations. Concurrently, there was an increase in the availability of onsite EMS physicians, indicating enhanced resource utilization. Procedurally, the analysis revealed a shift in diagnostic patterns, with a decline in teleconsultations for missions with tracer diagnoses and a corresponding increase in those without. This trend suggests an evolving landscape of EMS needs and capabilities. Furthermore, the study highlighted improvements in operational efficiency, notably evidenced by a reduction in the duration of teleconsultations. Additionally, the system’s capacity to handle multiple cases simultaneously was underscored, with approximately one-fourth of missions involving concurrent teleconsultations. In essence, the findings underscored the transformative impact of the tele-EMS system on EMS operations, as reflected by the observed structural enhancements and procedural refinements. These outcomes emphasize the pivotal role of telehealth in optimizing EMS performance and addressing evolving healthcare demands, as elucidated by the discerned KPIs.

The article by Vo et al. [32] proposes an Integrated Telehealth Model (ITM) within the framework of the Centers for Medicare and Medicaid Services’ meaningful use (MU) program. With a focus on KPIs, the model aims to incentivize the implementation of telehealth to enhance healthcare quality and efficacy. The ITM Incentive Program consists of three stages mirroring the MU program. Stage 1 incentivizes Medicaid providers to adopt telehealth technology, while Stage 2 introduces a tiered incentive system tied to increasing patient consultations. In Stage 3, providers are encouraged to sustain telehealth use by meeting clinical outcome objectives. The model aligns with the success of MU in promoting electronic health records (EHRs) adoption and leverages tiered incentives to drive telehealth integration. By addressing cost issues and emphasizing quality improvement, the ITM Model presents a strategic approach to enhancing healthcare delivery through technology, clinical processes, and integrated care.

Prag et al. in [33] highlights the necessity for a coordinated national approach in the United Kingdom to effectively manage chronic diseases and alleviate the burden on the National Health Service (NHS). It underscores the potential of telehealth systems to enhance healthcare delivery and quality while reducing economic strain. KPIs play a pivotal role in assessing the cost-efficiency of telehealth systems. However, measuring their impact on healthcare outcomes, such as reducing emergency room visits, unnecessary hospital visits, and enabling continuous vital signs monitoring for timely intervention, poses challenges both qualitatively and quantitatively.

The study by Al Dossary et al. [34] aims to identify and assess peer-reviewed publications on deployed telehealth services in hospital settings, focusing on the methodology used for evaluation. 164 papers were identified, representing 137 telehealth services, predominantly in the United States. Most services were real-time telehealth. About half of the studies evaluated services from clinical, economic, and satisfaction perspectives, also using specific KPIs, while the others described services without evaluation. Only a few studies indicated a structured implementation and evaluation process, with limited information on planning strategies. The review emphasizes the need for improved reporting of telehealth service implementation and planning strategies to facilitate learning and sustainable service development.

The work proposed by Islam et al. [35] a novel approach for video data transmission in 5G networks for telehealth applications, combining the H.265 protocol with a KNN classifier-based single buffer model and multiple sensors. The proposed method aims to improve data transmission efficiency and accuracy between transmitter and receiver devices. KPi metrics include collision error, propagation error, sensing error, and visual security with encryption. Comparative analysis with existing methods, particularly the Lagrangian Encoder-based single buffer, shows that the proposed KNN classifier-based single buffer with a multi-sensor technique outperforms existing approaches.

The study reported by Chang et al. [36] proposes a comprehensive evaluation framework for telehealth systems, emphasizing the importance of KPIs in assessing their effectiveness. Through a structured approach, KPIs related to information technology, service provider and consumer satisfaction, cost, quality, and information security are identified and organized within the framework. This framework enables stakeholders to make informed decisions based on evidence from KPIs, ensuring the quality and safety of telehealth services. Further research is needed to validate and enhance this framework across various contexts and participant groups, emphasizing the importance of KPIs in evaluating telehealth implementation.

The contribution by Larsen et al. [37] introduces a groundbreaking concept: a “shared service center” designed to bolster the implementation and widespread adoption of telehealth initiatives. Developed through a year-long participatory process involving diverse stakeholders, this center aims to provide essential support services. Through collaborative efforts, stakeholders identified four key service categories crucial for the success of telehealth endeavors. Importantly, this initiative underscores the significance of KPIs in evaluating and ensuring the effectiveness and efficiency of telehealth implementations. By emphasizing KPIs, stakeholders seek to establish a framework for assessing the impact and success of telehealth initiatives, ultimately advancing the quality and reach of healthcare services.

Zimmer-Galler et al. [38] focuses on the application of telehealth in Ophthalmology. They remarked that telehealth holds promise in addressing challenges related to diabetic retinopathy screening, aiming to improve key performance indicators (KPIs) such as screening adherence and patient outcomes. Despite evidence-based guidelines, many diabetic patients do not undergo recommended annual retinal evaluations, leading to vision loss. Telehealth offers a solution by increasing screening rates and reducing complications. Studies demonstrate the efficacy of telehealth programs in large population-based systems, with emerging evidence from community-based initiatives supporting their positive impact on KPIs. New imaging platforms enhance detection and grading, but financial barriers persist. Overall, telehealth presents a viable approach to mitigate the healthcare burden of diabetic retinopathy, with KPIs guiding program evaluation and resource allocation.

### 3.2. Outcome from the Analysis of Institutional WEBs

Alongside the analysis of scientific literature, examining documents produced by international organizations and associations provides a key perspective on established practices and emerging trends in the field of telehealth. In this context, a relevant document is that of the World Health Organization (WHO) [39], which, with its focus on disability, fragility, and disadvantaged populations, positions telehealth as a crucial tool to ensure equitable access to healthcare services. Subsequently, the analysis extends to two documents from the United States. The first document, made available among the online documents by the ATA by the American Telemedicine Association [40], represents an important reference point, being ATA the most authoritative association in telehealth. It provides a holistic approach to telehealth, reflecting the broad scope of this discipline. The second document, produced by the American College of Physicians [41], (the largest medical organization specializing in internal medicine, specialized in producing clinical guidelines, evidence-based recommendations, and educational resources for internists and other healthcare providers) reflects the orientation towards internists in the adoption and effective use of telehealth in their clinical practice. The analysis then continues with a document from the United Kingdom National Health Service (NHS) [42], which focuses on the application of telehealth in the context of Integrated Urgent Care. This document offers an interesting perspective on the practices and specific challenges encountered in the context of the United Kingdom. Finally, the analysis of the document produced by the health authority of Dubai [43] provides an insight into ongoing initiatives in emerging economies, which are significantly investing in telehealth services. This document is particularly important as it shows how telehealth is becoming a priority even in contexts not traditionally associated with such healthcare innovations. The WHO has been chosen as a supra-national entity that is impartial. The American Telemedicine Association and the American College of Physicians have been selected as respectively the most important telehealth association in the world and the most important medical association in the world. The NHS document from the British Healthcare system [42] has been chosen because it is a contribution from the old Western world. The Dubai document represents a contribution from an emerging country with rapid economic development that is heavily investing in telehealth.

Overall the documents reflect both the role of the institution and the specific characteristics of the region where telehealth is implemented. This includes how the institution’s policies and practices are shaped by and adapt to the unique needs and conditions of the local population, as well as the impact of telehealth on healthcare delivery within that particular area. By considering these factors, the documents provide a comprehensive view of how telehealth functions in diverse settings, highlighting the interplay between institutional frameworks and local realities.

The following paragraphs delve into detail on each contribution.

#### 3.2.1. The Position of the WHO

The WHO document [39] on telehealth KPIs highlights the importance of demonstrating benefits to address the challenges that healthcare service providers face in change [39]. Several evaluation indicators are suggested, divided into three temporal categories: short term, medium term, and long term.

Short Term
  - Increase in the number of specialties per unit offered
  - Increase in teleconsultations conducted
  - Patient savings
  - Time elapsed between teleconsultation request and actual execution
  - Satisfaction questionnaire
  - Waiting time for teleconsultation
  - Number of teleconsultations in a given period
Medium Term
  - Ratio of teleconsultations conducted to corresponding decrease in regular consultations
  - Percentage of hospitals adopting telehealth compared to the national total
  - Number of deferred teleconsultations
  - Number of unused or free hours
  - Number of technical issues per unit
  - Time taken to resolve technical issues per unit
Long Term
  - Number or percentage of patients monitored via telehealth
  - Improvement in monthly consultations compared to previous years
  - Average savings compared to the previous year

The document also emphasizes in relation to the KPis the importance of evaluating various aspects of telehealth projects from the outset and proposes a series of effectiveness, quality, and efficiency indicators to be used during all project phases. Participants in the document also suggest that the frequency of indicator measurement depends on the project phases and the specific situation, and that the implementation of new telehealth projects should be officially incorporated into the country’s information systems. The importance of measuring the impact on health, the effectiveness of telehealth projects, and identifying savings resulting from such projects is highlighted. Furthermore, the document underscores the importance of monitoring and evaluation in telehealth, reiterating that this process should be present from the beginning of the project. It is suggested to evaluate various aspects both individually and collectively, including macro aspects such as sociocultural, economic, political, legal, technological infrastructure, and R&D considerations, as well as ecological considerations. Sectoral aspects such as providers, competitors, substitutes, entry barriers, market forces, the role of academia, medical associations, regulators, acceptability for patients and communities, the role of telecommunications companies, interoperability and scalability in the healthcare system, and pre-hospital development, among others, are also included. Micro aspects such as strengths, weaknesses, opportunities, threats, financial, technical, political, and social feasibility, and social responsibility are suggested to be evaluated. Process indicators such as acceptability, technological scalability, continuity of care, and responsiveness capacity are suggested to be monitored before, during, and at the end of the project. Priority in evaluation depends on the project stage: in the initial stages, it should focus on achieving initial objectives and aligning strategies with targets, while in later stages, it should optimize processes and technologies, and finally evaluate social and economic benefits. It is suggested to systematically evaluate whether proposed objectives have been achieved, using, for example, the Balanced Scorecard to develop indicators. Indicators should be clear, precise, comparative, and measured at a certain frequency. The measurement frequency may vary depending on circumstances, and the evaluation team’s knowledge of the project is essential to make any changes. To incorporate new projects into official sources, clear definitions of what will be measured, evidence supporting the information to be measured, agreements with the authorities responsible for the country’s information systems, and operational consensus on the format and variables to be measured are suggested. Finally, it is suggested to measure the health impact, using representative indicators such as access to healthcare services, perception of quality and benefits of telehealth, and indicators based on the health economics framework to identify savings. Primary indicators are also proposed for telehealth programs, including economic indicators and for specific services such as tele-education. The document includes useful sheets for reporting these indicators.

#### 3.2.2. The Document Provided by ATA

The document in [40] emphasizes the importance of balancing clinical excellence with operational efficiency, highlighting that telehealth presents new opportunities but can complicate the measurement of care quality between remote and in-person settings. It underscores the significance of patient experience in both environments and the necessity of integrating business systems to maintain a consistent experience. Several key challenges are identified in this context.

According to the document, these challenges include:

- Limited understanding of how to deliver the same or better quality of care in a remote setting compared to a physical one.
- Continuous need to accurately identify and monitor high-risk patients and unplanned hospital visits.
- Limited comprehension of patient behavior details and the reasons for telehealth visits.
- Growing concern over the need for data and its impact on patient privacy.

To address these challenges, a solution framework is proposed that includes:

- Creation of configurable clinical dashboards to monitor large groups of patients and actively identify high-risk patients.
- Generation of real-time quality assurance reports to better assess the quality of care.
- Determination of KPIs related to patient location and outcomes to track longitudinal patient movement through the healthcare system.
- Utilization of provider dashboards to better understand individual performance metrics and efficiency.
- Provision of data-driven feedback on patient usage to optimize resources and increase patient engagement and satisfaction.

The document also emphasizes the importance of interoperability and data integration among various telehealth solutions, as well as the need to monitor and track data in real-time to optimize operational and financial efficiency and secure timely reimbursement. Furthermore, it highlights the necessity of a strategic vision and proactive investment in data aggregation solutions and integrated platforms to support business, operational, and clinical decisions in the telehealth sector.

Before detailing the KPIs, the document underscores the growing demand for telehealth and its impact on healthcare systems and financial outcomes. It notes that while challenges related to reimbursement are decreasing, healthcare systems must adapt to capture and track the data needed for compliance with evolving payment reporting requirements. Additionally, it points out that integrated telehealth has the potential to reduce costs, but it is essential to capture the correct data at the right points along the patient journey to optimize programs aimed at achieving this goal. The document then presents 15 KPIs grouped into the following categories:

Clinical:

1. Patient Prioritization: Effectiveness in identifying and treating high-risk patients.
2. Measurement of clinical efficiencies to optimize processes so providers can reach patients more quickly and frequently.
3. Comparative success factors in defining care quality across different service lines.
4. Distribution of referral facilities: Ability to transfer patient data between environments based on required specialist care.
5. Patient Behavior: Reasons for telehealth visits.

Operational/Technological:

1. Efficiency and effectiveness of telehealth services.
2. Accuracy of collected data.
3. Collection of technical data in addition to clinical data (defect-free encounters, etc.).
4. Effectiveness in resource allocation.
5. Quality definitions across service lines.

Administrative/Executive:

1. Monitoring telehealth visits and completion rates.
2. Revenue generated per service line and care modality.
3. Effectiveness in mitigating penalties for repeat hospitalizations.
4. Success in resource allocation.
5. Turnover of involved patients: Loss or gain of patients.

In summary, these KPIs aim to evaluate clinical effectiveness, operational efficiency, resource management, and financial sustainability of telehealth services. Collecting and analyzing this data is crucial for guiding business decisions and improving the overall quality of healthcare delivered through telehealth.

#### 3.2.3. The Position of the ACP

The American College of Physicians (ACP) document is a position paper [41] that offers recommendations for developing KPIs for telehealth. The recommendations were developed by the ACP Performance Measurement Committee, which reviewed various sources including studies and reports on trends, adoption of telehealth, and patient and physician satisfaction. Sources older than five years were excluded to reflect recent data from the pandemic. The document underscores several key points:

- Quality standards for telehealth are still in their early stages.
- The same general principles of quality measurement applied to in-person care should be applied to telehealth to ensure consistent quality without negatively affecting clinical outcomes.
- Performance measures should be evaluated and adjusted to include telehealth care.
- Each performance measure should be tested for its reliability, validity, and attribution in a telehealth environment.
- A significant limitation in healthcare remains the availability and accessibility of data, which must be particularly addressed for telehealth visits.
- Any performance measure should be carefully evaluated to avoid unintended consequences, especially negative impacts on disadvantaged communities.

The document provides specific recommendations, as follows:

1. Recommendation 1: Performance measures used to evaluate the quality of care provided by a physician during a telehealth visit should follow the same principles and criteria as those for an in-person outpatient visit.
2. Recommendation 2: Performance measures should be assessed to determine whether the care provided during a telehealth visit should be included in the specifications, with careful consideration of how this inclusion might affect the measure or lead to unintended consequences.
3. Recommendation 3: Mechanisms should be implemented to allow physicians and their information systems to access information generated during a telehealth visit before a performance measure is used to evaluate the quality of care.
4. Recommendation 4: Performance measures should be tested for telehealth visits before being used to evaluate the quality of care provided by a physician.
5. Recommendation 5: Telehealth visits should be incorporated into the attribution logic of the measures, considering whether they should be attributed to a specific physician, a medical group, or a health plan.
6. Recommendation 6: Performance measures for telehealth should not discriminate against disadvantaged communities already affected by the digital divide.

#### 3.2.4. The Position of the English National Health Service

The NHS document [42] details the KPIs related to Integrated Urgent Care (IUC) activities. This document is particularly interesting and useful as it also addresses remote interaction through telehealth during these activities, thus offering a complementary perspective. It comprehensively outlines the KPIs to be applied by the stakeholders involved in this service. It is intended for use by the entities involved in service delivery, providers, and the NHS. Including telehealth as an integral part of integrated urgent care activities highlights the importance of this approach in optimizing healthcare delivery and ensuring efficient and effective access to healthcare services for patients.

Here are the KPIs from the document:

1. Proportion of abandoned calls: Measures the percentage of abandoned calls, with a maximum abandonment standard of 3%.
2. Average call response time: Measures the average time taken to answer calls, with a maximum standard of 20 s.
3. 95th percentile call response time: Measures the call response time at the 95th percentile, with a maximum standard of 120 s.
4. Proportion of calls assessed by a doctor or clinical advisor: Measures the percentage of calls assessed by a doctor or clinical advisor, with a minimum threshold of 50%.
5. Proportion of calls assessed by a doctor within an agreed timeframe: Measures the percentage of calls assessed by a doctor within an agreed timeframe, with a minimum threshold of 90%.
6. Proportion of callers recommended for self-care at the end of the clinical intervention: Measures the percentage of calls where callers were recommended for self-care at the end of the clinical intervention, with a minimum threshold of 15%.
7. Proportion of calls initially assigned to a category 3 or 4 ambulance disposition receiving remote clinical intervention: Measures the percentage of calls initially assigned to a category 3 or 4 ambulance disposition receiving remote clinical intervention, with a minimum threshold of 75%.
8. Proportion of calls initially assigned to an ETC disposition receiving remote clinical intervention: Measures the percentage of calls initially assigned to an ETC disposition receiving remote clinical intervention, with a minimum threshold of 50%.
9. Proportion of calls assigned the first type of service offered by the Directory of Services: Measures the percentage of calls assigned the first type of service offered by the Directory of Services, with a minimum threshold of 80%.
10. Proportion of calls scheduled for an appointment at a general practice or a GP access hub: Measures the percentage of calls scheduled for an appointment at a general practice or a GP access hub, with a minimum threshold of 75%.
11. Proportion of calls scheduled for an appointment at an IUC Treatment Service or the caller’s residence: Measures the percentage of calls scheduled for an appointment at an IUC Treatment Service or the caller’s residence, with a minimum threshold of 70%.
12. Proportion of calls scheduled for an appointment at an Urgent Treatment Centre (UTC): Measures the percentage of calls scheduled for an appointment at an Urgent Treatment Centre (UTC), with a minimum threshold of 70%.
13. Proportion of calls assigned a booked time slot with a Type 1 or 2 Emergency Department: Measures the percentage of calls assigned a booked time slot with a Type 1 or 2 Emergency Department, with a minimum threshold of 70%.
14. Proportion of calls scheduled for an appointment with a Same Day Emergency Care (SDEC) service: Measures the percentage of calls scheduled for an appointment with a Same Day Emergency Care (SDEC) service.

#### 3.2.5. The Position of the Dubay Health Athority

The document titled “Guidelines for Reporting Telehealth Key Performance Indicators” [43] was issued by the Health Regulation Sector (HRS) of the Dubai Health Authority (DHA). It was published on 22 August 2021 and came into effect on 22 October 2021. This document outlines the procedures and standards for reporting KPIs related to telehealth. The introduction emphasizes that the HRS is tasked with developing regulations, policies, standards, and guidelines to enhance patient quality and safety and promote the growth and development of the healthcare sector. The guidelines aim to achieve the strategic objectives of the Dubai Health Authority, including positioning Dubai as a global medical destination and ensuring a happy, healthy, and safe environment for Dubai’s population.

The document defines telehealth as the use of virtual technology and telecommunications to deliver healthcare services outside traditional healthcare facilities and without physical visits. It provides a set of KPIs divided into two categories: Access and Quality. Some examples of KPIs include patient waiting time percentage, percentage of referrals to in-person consultations with doctors or specialists, and percentage of emergency telehealth calls, among others. The document also provides an overview of the general procedures for collecting and reporting KPI data, as well as the deadlines for quarterly data reporting. It highlights that all DHA-licensed healthcare facilities providing telehealth services must regularly collect and report the specified KPI data using the provided tools.

The KPIs are organized into two groups (Access and Quality), with suggested reporting forms for each KPI.

Access:

1. Percentage of Patient Waiting Time (days): Measures the percentage of patient waiting time in days, assessing the efficiency and timeliness in delivering telehealth services.
2. Percentage of In-Person Referrals by Doctors, Specialists, or Consultants: Measures the percentage of patients referred for an in-person consultation with a doctor, specialist, or consultant, evaluating the need for further assessment or treatment.
3. Percentage of Emergency Referrals in Telehealth: Measures the percentage of patients referred to emergency services following a telehealth consultation, assessing the management of emergencies through this channel.
4. Percentage of Telehealth Calls from Outside Dubai: Measures the percentage of telehealth calls received from individuals outside the Dubai area, indicating the geographical reach of telehealth services.
5. Population Coverage (Consultation) by Location: Measures the percentage of the population served by telehealth consultations based on geographical location.
6. Population Coverage (Tele-Prescription) by Location: Measures the percentage of the population served by telehealth prescriptions based on geographical location.
7. Population Coverage (Tele-Monitoring) by Location: Measures the percentage of the population served by remote health condition monitoring through telehealth based on geographical location.

*Quality:*

8. Percentage of Followed Patients (High-Risk Groups): Measures the percentage of high-risk patients regularly monitored through telehealth services, assessing the effectiveness in monitoring and managing such patient groups.
9. Percentage of Medication Prescriptions via Teleconsultation: Measures the percentage of patients receiving medication prescriptions through telehealth consultations, evaluating the appropriateness and safety of prescriptions made through this channel.
10. Percentage of Antibiotic Prescriptions via Teleconsultation: Measures the percentage of patients receiving antibiotic prescriptions through telehealth consultations, evaluating the appropriateness of antibiotic use in this context.
11. Percentage of Sick Leave Prescriptions via Teleconsultation: Measures the percentage of patients prescribed sick leave through telehealth consultations, assessing the management of health-related absences through this channel.
12. Percentage of Medication Prescription Errors Related to Teleconsultation: Measures the percentage of medication prescription errors during telehealth consultations, highlighting potential patient safety risks.
13. Percentage of Patient Satisfaction in Telehealth: Measures the percentage of patients expressing satisfaction with the telehealth services received, assessing the overall patient experience and satisfaction with the care provided.
14. Percentage of Staff Satisfaction in Telehealth: Measures the percentage of healthcare providers expressing satisfaction with the telehealth services provided, also evaluating staff engagement and satisfaction in delivering such services.
15. Percentage of Complaints Against Telehealth Services: Measures the percentage of complaints received regarding telehealth services, highlighting any concerns or issues raised by users or staff.

## 4. Discussion

Our overview focuses on the introduction of Key Performance Indicators (KPIs) in telehealth as a strategic tool to enhance its integration within the healthcare domain. A key added value of our study, compared to other studies of this type, is that it does not address a specific aspect or sector of telehealth, as seen in references [22,23,24], but rather delves into the broad field of telehealth. Another significant added value is that it incorporates two perspectives; in addition to the first perspective based on scientific literature, similar to previous studies [21,34], it introduces a second perspective focused on the positions of relevant institutions in this field. This approach balances the authors’ viewpoint with the positions of relevant entities and authorities in this area.

In the discussion, the emerging highlights from both perspectives are detailed separately and then connected to interpret their complementary contributions.

The first section (Section 4.1) discusses the evidence emerging from the perspective of scientific studies, focusing on both the opportunities and the areas that need further development. The second section (Section 4.2) applies the same discussion approach to the evidence emerging from institutional documents. The third section (Section 4.3) bridges the two perspectives and offers recommendations. This is followed by the limitations (Section 4.4) and the takeaway message (Section 4.5).

### 4.1. Highlights from the Point of View Focused on the Scientific Literature

The overview has shown both significant opportunities and challenges and limitations in this specific field.

Opportunities

The integration of KPIs in telehealth offers numerous opportunities for enhancing healthcare delivery and improving patient outcomes [21,22,23,24,25,26,27,28,29,30,31,32,33,34,35,36,37,38]. By evaluating various metrics such as response times, problem resolution rates, appointment adherence, consultation durations, and patient satisfaction, healthcare providers can identify areas for improvement and streamline processes. Additionally, KPIs play a crucial role in assessing the cost-effectiveness of telehealth programs, ensuring regulatory compliance, and facilitating data-driven decision-making to continuously enhance service delivery [21,32,38] in different fields, from pharmacy [24] up to emergency medical care [31] or chronic care [38] and show an important impact in the telehealth integration [32,33,34,35] (Table 2).

One significant opportunity lies in leveraging KPIs to monitor and enhance the effectiveness of remote healthcare services [22]. Through continuous monitoring of KPIs, healthcare providers can identify trends, patterns, and areas for improvement, thereby optimizing telehealth service delivery. Moreover, KPIs enable healthcare organizations to ensure high-quality, patient-centered care by focusing on key aspects of service provision, such as clinical outcomes and patient satisfaction [22].

Furthermore, the use of KPIs in telehealth allows for the measurement of clinical outcomes and the evaluation of program effectiveness [23]. By tracking KPIs related to clinical outcomes, such as disease management and patient recovery rates, healthcare providers can assess the impact of telehealth interventions on patient health and well-being. This enables them to make data-driven decisions regarding the allocation of resources and the implementation of interventions that are most effective in improving patient outcomes [23]. Moreover, KPIs help in ensuring regulatory compliance and adherence to quality standards in telehealth practice [24]. By tracking KPIs related to regulatory requirements and quality metrics, healthcare organizations can demonstrate compliance with relevant guidelines and standards, ensuring the delivery of safe and effective telehealth services [24]. Additionally, the use of KPIs facilitates benchmarking and performance comparison across different telehealth programs and providers [25]. By establishing standardized KPIs and performance metrics, healthcare organizations can compare their performance with industry benchmarks and identify areas for improvement. This enables continuous learning and improvement, driving innovation and excellence in telehealth practice [25]. Overall, the integration of KPIs in telehealth offers numerous opportunities for enhancing healthcare delivery, improving patient outcomes, and driving continuous improvement in service quality and efficiency [21,22,23,24,25,26,27,28,29,30,31,32,33,34,35,36,37,38].

Areas needing further efforts

Based on the overview, further efforts are needed in several areas related to the development and utilization of KPIs in healthcare, particularly in the context of digital health interventions (DHIs) and telehealth [21,22,23,24,25,26,27,28,29,30,31,32,33,34,35,36,37,38]. Here are the main areas requiring attention:

- Standardization of Methodologies for KPI Development:
  Brenner et al. [21] highlighted a significant gap in evidence-based knowledge on KPI development for DHIs and a lack of standardized methodologies. Further research is needed to establish common methods for KPI development to improve comparability and advance the field.
- Comprehensive Assessment of Telehealth Impact:
  All the studies [21,22,23,24,25,26,27,28,29,30,31,32,33,34,35,36,37,38] and in particular details those by Duong et al. [22] and Caffery et al. [23] underscore the importance of utilizing KPIs to comprehensively assess the impact of healthcare innovations like telehealth on patient care pathways. There’s a need for more research to identify and develop KPIs that accurately capture the effects of telehealth on various aspects of healthcare delivery.
- Consensus on Outcome Measures and Quality Indicators:
  Canning et al. [24] emphasized the need to establish consensus on outcome measures and quality indicators for clinical pharmacy services. Further efforts are required to develop standardized sets of KPIs that can be universally applied to assess the quality and effectiveness of clinical pharmacy services across different healthcare settings.
- Evaluation Frameworks for Telehealth Systems:
  Chang et al. [36] proposed a comprehensive evaluation framework for telehealth systems, but further research is needed to validate and enhance such frameworks across various contexts and participant groups. This includes identifying additional KPIs and refining evaluation methods to ensure the quality and safety of telehealth services.
- Improvement of Reporting Standards in Telehealth Studies:
  Al Dossary et al. [34] highlighted the need for improved reporting of telehealth service implementation and planning strategies to facilitate learning and sustainable service development. Efforts should focus on enhancing reporting standards to ensure transparency, reproducibility, and comparability of telehealth studies, including the use of KPIs.
- Addressing Challenges in Disease Management:
  Prag et al. [33] stressed the necessity for a coordinated national approach to effectively manage chronic diseases using telehealth systems. Future research should focus on developing KPIs that accurately measure the impact of telehealth on healthcare outcomes, including reducing emergency room visits, unnecessary hospital visits, and enabling continuous vital signs monitoring for timely intervention.

By addressing these areas, researchers and healthcare professionals can advance the development and implementation of KPIs in healthcare, particularly in the context of digital health interventions and telehealth, ultimately leading to improved patient care and outcomes.

### 4.2. Highlights from the Point of View Focused on the National and International Institutions

First and foremost, it is necessary to clarify that the analysis at this point did not aim to naturally identify one standout entity over another in identifying KPIs. Rather, its goal was to identify, on one hand, a sample useful for providing a general perspective on this issue (a supra-governmental entity: WHO; two prominent international companies: ATA and ACP; a European healthcare entity: NHS; and a rapidly growing healthcare entity from an emerging country: Dubai Health Authority). On the other hand, it aimed to identify entities that directly and explicitly provide easily accessible information on KPIs. It is important to note and reaffirm how other significant entities are addressing these issues in a structured manner. For example, the International Society for Telemedicine and eHealth (ISfTeH) [44], a well-known international organization focused on telehealth, indirectly addresses these issues through documents such as the “2018-19-INTERNATIONAL-TELEHEALTH-CODE-OF-PRACTICE”(even if not directly on the KPIs) available on their website [45]. Similarly, the European Connected Health Alliance (ECHAlliance) [46], in documents like the one referenced in [47], addresses these themes and highlights the procedural importance of using KPIs (even if not face the KPis specifically). Recommendations [47] include defining accurate performance measures and establishing monitoring mechanisms, agreeing internally on how to assess the success of pilot projects, and if possible, using outcome-based KPIs that can serve as scientific evidence. Additionally, creating clear operational guidelines is advised, specifying the course of action if the defined KPIs are achieved by the end of the pilot phase, clarifying the level of commitment from decision-makers to adopt the pilot, determining internal ownership of the initiative thereafter, and outlining long-term funding mechanisms for healthcare organizations. It is also important to consider the potential contribution in this field from The Digital Health Society (DHS), as part of the ECHAlliance Group, which facilitates multi-stakeholder dialogue on digital health and health data policies and practices [48].

Table 3 reports a sketch of the potential connections of these three international societies with the theme of the KPI

The direct focus on the examined documents from both national and international entities has not only provided a more comprehensive perspective but has also revealed a diverse and multifaceted focus. This diversity, while distinct, contributes to a more holistic understanding when considered collectively [39,40,41,42,43]. Such varied focuses are influenced by the roles and functions of different institutions within the healthcare landscape.

Emerging focus

The analysis of KPIs from various sources sheds light on particularly distinct focuses regarding telehealth practices and their evaluation. The WHO document [39] emphasizes the importance of demonstrating the benefits of telehealth to address challenges in healthcare service provision during periods of change. It suggests evaluation indicators categorized into short term, medium term, and long term, covering aspects such as the increase in teleconsultations, patient savings, and the number of patients monitored via telehealth. On the other hand, the document provided by ATA [40] stresses the need to balance clinical excellence with operational efficiency in telehealth. It identifies key challenges like understanding how to deliver quality care remotely and accurately monitoring patient behavior. The document proposes solutions like creating configurable clinical dashboards and generating real-time quality assurance reports to address these challenges effectively.

Similarly, the document from the ACP [41] offers recommendations for developing KPIs for telehealth, emphasizing the importance of applying quality measurement principles from in-person care to telehealth. It highlights the need for reliable, valid, and attributable performance measures tailored to telehealth environments and underscores the importance of avoiding unintended consequences, particularly negative impacts on disadvantaged communities. Furthermore, the NHS document [42] outlines KPIs related to Integrated Urgent Care activities, incorporating telehealth as an integral part of optimizing healthcare delivery. It provides a comprehensive set of KPIs for stakeholders involved in IUC services, focusing on metrics like abandoned calls, call response times, and the proportion of calls assessed by a doctor within an agreed timeframe.

Lastly, the guidelines from the Dubai Health Authority [43] establish procedures and standards for reporting telehealth KPIs, aiming to enhance patient quality and safety and promote the growth of the healthcare sector in Dubai. These guidelines categorize KPIs into Access and Quality, covering aspects such as patient waiting time, population coverage, medication prescriptions, patient satisfaction, and staff satisfaction. Overall, these documents offer valuable insights into the evaluation of telehealth practices, highlighting the importance of demonstrating benefits, balancing clinical excellence with operational efficiency, applying quality measurement principles, incorporating telehealth into existing healthcare frameworks, and ensuring patient safety and satisfaction.

Table 4 synthetizes the different focus on the KPIs of each one of the analysed entities.

Emerging perspectives and priorities

In addition to highlighting common themes and approaches to evaluating telehealth practices, the documents also underscore diverse perspectives and priorities shaped by the contexts of different healthcare systems and regions. The documents in fact reflect both the role of the institution and the specific characteristics of the region where telehealth is practiced. They highlight how institutional policies adapt to local needs and conditions, demonstrating the interplay between the institution’s framework and the realities of the area served by telehealth.

For instance, the WHO document [39] emphasizes equitable access to healthcare services, particularly for disadvantaged populations, positioning telehealth as a crucial tool to address disparities. This perspective reflects a global health focus on social determinants of health and the need to ensure healthcare access for all. On the other hand, the document provided by ATA [40] emphasizes the challenges of balancing clinical excellence with operational efficiency in telehealth, reflecting the perspective of a leading telehealth association focused on optimizing the delivery of care while maintaining quality standards. The ACP document [41] addresses the need to develop tailored performance measures for telehealth while considering the potential impact on disadvantaged communities. This reflects a concern for equity and inclusivity in telehealth initiatives, particularly in diverse socio-economic contexts.

The NHS document [42] focuses on KPIs related to Integrated Urgent Care activities, including telehealth, highlighting the specific challenges and priorities within the UK national healthcare system. This perspective underscores the importance of integrating telehealth into broader healthcare delivery frameworks tailored to regional needs.

Finally, the guidelines from the Dubai Health Authority [43] reflect the priorities and objectives of the Dubai healthcare sector, emphasizing the importance of reporting telehealth KPIs to enhance patient quality and safety while promoting Dubai as a global medical destination. This perspective highlights the strategic objectives of a rapidly developing healthcare system in an emerging economy. Overall, these diverse perspectives illustrate the multifaceted nature of telehealth evaluation, shaped by regional priorities, healthcare system characteristics, and socio-economic contexts. By understanding and incorporating these diversities, stakeholders can develop more effective telehealth strategies tailored to specific needs and challenges.

### 4.3. Joint Interpretation from Two Perspectives

When we integrate insights from both scientific studies [21,22,23,24,25,26,27,28,29,30,31,32,33,34,35,36,37,38] and institutional documents [39,40,41,42,43] regarding KPIs in telehealth, we gain a comprehensive understanding of the metrics used to assess the effectiveness, efficiency, and quality of telehealth services. Scientific studies provide empirical evidence and theoretical frameworks for understanding the impact of telehealth on healthcare delivery [21,22,23,24,25,26,27,28,29,30,31,32,33,34,35,36,37,38]. They analyze various aspects such as patient outcomes, cost-effectiveness, provider satisfaction, and technological feasibility. These studies contribute valuable insights to the development of KPIs by identifying indicators that correlate with positive outcomes or highlight areas needing improvement.

On the other hand, institutional documents from international organizations like the WHO [39] and professional associations like the [40] offer guidelines and recommendations for implementing telehealth practices. These documents often outline specific KPIs tailored to different contexts and healthcare settings, reflecting best practices and industry standards. For instance, the WHO document emphasizes the importance of patient access and equity, while the ATA document focuses on balancing clinical excellence with operational efficiency.

By synthesizing findings from both scientific studies and institutional documents, we can create a robust framework for evaluating telehealth initiatives. Scientific evidence informs the selection of KPIs based on their proven impact on patient care and operational efficiency, while institutional guidelines ensure that KPIs align with industry standards and regulatory requirements. This integrated approach facilitates continuous improvement and optimization of telehealth services, promoting a dynamic feedback loop between research and practice.

Emerging recommendations

In summary, the joint interpretation of scientific studies [21,22,23,24,25,26,27,28,29,30,31,32,33,34,35,36,37,38] and institutional documents [39,40,41,42,43] on KPIs in telehealth provides a comprehensive framework for assessing the effectiveness, efficiency, and quality of telehealth services, thereby facilitating continuous improvement and optimization of telehealth initiatives. The fusion of insights from scientific studies [21,22,23,24,25,26,27,28,29,30,31,32,33,34,35,36,37,38] and institutional documents [39,40,41,42,43] offers valuable guidance for both scholars and stakeholders in telehealth. Key recommendations include developing comprehensive frameworks, prioritizing evidence-based KPIs, ensuring regular evaluation, fostering interdisciplinary collaboration, maintaining transparency and accountability, adapting to change, allocating resources effectively, and promoting knowledge sharing.

By embracing these recommendations, *stakeholders* can enhance the effectiveness and efficiency of telehealth initiatives, ultimately improving healthcare delivery and outcomes. 

For scholars, the synthesis of scientific studies and institutional documents provides a rich foundation for further research and analysis in telehealth. They can leverage existing literature and institutional insights to deepen their understanding of telehealth practices, challenges, and opportunities. Scholars should focus on filling knowledge gaps, conducting empirical studies to validate proposed KPIs, exploring emerging trends, and advancing theoretical frameworks. Additionally, they should disseminate their findings through peer-reviewed publications, conferences, and policy briefs to contribute to the scholarly discourse and inform evidence-based decision-making by stakeholders. By engaging in rigorous research and knowledge dissemination, scholars can contribute to the advancement of telehealth as a critical component of modern healthcare delivery.

### 4.4. Takeway Message

The integration of scientific studies and institutional documents on telehealth KPIs underscores the importance of evidence-based decision-making in healthcare. Stakeholders must leverage existing knowledge to optimize telehealth practices, address challenges, and maximize benefits. Scholars play a crucial role in advancing research, validating KPIs, and informing policy and practice. By synthesizing insights from diverse sources, stakeholders can ensure the effective implementation and continuous improvement of telehealth services, ultimately enhancing access to quality healthcare for all.

### 4.5. Limitations

The study was conducted through searches in scientific databases for scientific studies and on the web for documents from international and national institutions. Narrative review has the potential to bring out the elements and themes at play concerning scientific studies. Targeted systematic reviews can delve specifically into emerging themes. However, the web search for institutional documents has limitations due to the online sharing options that some institutions may not have chosen. To be honest, the web search did not reveal any specific documents from the European community. This reflects the fact that the healthcare systems of the various member countries have their own different visions of their respective healthcare systems. Although based on the concept/approach of universality in healthcare provision, they are architecturally structured in different ways. In our case, the search for documents and the selection according to a justified rationale (*head of paragraph 3.2*) highlighted the different focus and complementarity of the approach. Targeted investigations on each institution, also considering non-online documents, may lead to the realization of other particularly specialized scientific studies. Furthermore, conducting targeted investigations into additional international societies such as ISfTeH, DHS, and ECHAlliance [44,45,46,47,48], along with exploring non-online accessible documents through specialized searches, could uncover further specialized scientific studies. These organizations play critical roles in the advancement of telemedicine and digital health, offering insights into cutting-edge practices, technological innovations, and policy frameworks. Accessing such documents, which may not be readily available through conventional academic databases or repositories, could reveal in-depth scientific research, case studies, or policy papers that provide perspectives on KPIs in telehealth. 

## 5. Conclusions

This study provides an overview of telehealth KPIs derived from scientific literature and institutional documents. Scientific studies emphasize KPIs for assessing telehealth effectiveness, efficiency, and quality, focusing on patient outcomes, operational efficiency, technical reliability, and cost-effectiveness. Institutional documents from global and national bodies like WHO, ATA, ACP, NHS, and DHA reflect regional telehealth policies, highlighting KPIs tailored to local healthcare needs. Key findings from scientific studies include the emphasis on patient health outcomes, operational efficiency metrics, technical reliability, and economic benefits. Institutional documents emphasize equitable access to healthcare, clinical excellence, and quality standards in telehealth, with specific KPIs ranging from patient access to clinical outcomes. Recommendations for scholars include conducting systematic reviews, expanding research to include non-online and unpublished sources, and promoting interdisciplinary studies. Stakeholders are advised to adopt comprehensive KPIs covering clinical outcomes, operational efficiency, and patient satisfaction, utilize international insights for policy development, invest in technology infrastructure, and continuously monitor and evaluate telehealth programs. Ultimately, integrating telehealth effectively into healthcare systems requires a holistic approach involving diverse KPIs from scientific research and institutional guidelines to enhance service effectiveness, efficiency, and equity in healthcare delivery.

## Figures and Tables

**Table 1:** Sketch on the focus on the KPI in each one of the overviewed study.

Study Reference	Focus of the Study	Focus on the KPI
[21]	Identifying current knowledge and evidence on developing KPIs for digital health interventions (DHIs)	Emphasizing stakeholder involvement in KPI development, highlighting the need for standardized methodologies
[22]	Assessing the effectiveness of teledermatology in healthcare	Utilizing KPIs, particularly time, to evaluate pathway efficiency
[23]	Evaluation of telehealth substitution rates	Telehealth-eligible rates, overall telehealth substitution rates
[24]	Consensus on KPIs for clinical pharmacy services	Relevance and measurability of clinical pharmacy quality indicators, patient outcome measures
[25]	Relationship between MPR-CQI implementation and patient survival in end-stage kidney disease	KPI target achievements, mortality reduction
[26]	Quantification of evaluation criteria in telehealth	Clinical outcomes, satisfaction, patient quality, cost measures
[27]	Comparison of quality of care between telehealth and in-person office visits	Health Care Effectiveness Data and Information Set (HEDIS) performance measures
[28]	Analysis of factors associated with telehealth implementation	Research performance, training, collaboration, legislative frameworks
[29]	Development and evaluation of a 5G usability test framework for preclinical diagnostics	Ultrasound image quality, transmission latency, data throughput
[30]	Assessment of the face validity of the Model for Assessment of Telehealth (MAST)	Importance of MAST domains and topics in relation to the KPIs
[31]	Longitudinal effects of integrating a tele-EMS system	Structural and procedural metrics, resource utilization, operational efficiency
[32]	Proposal of an Integrated Telehealth Model (ITM)	Incentive program stages, telehealth integration
[33]	Necessity for coordinated national approach to manage chronic diseases	Cost-efficiency of telehealth systems
[34]	Identification and assessment of peer-reviewed publications on telehealth services	Evaluation methodology, planning strategies
[35]	Introduction of a novel approach for video data transmission in 5G networks	Collision error, propagation error, sensing error, visual security
[36]	Proposal of a comprehensive evaluation framework for telehealth systems	Information technology, service provider and consumer satisfaction, cost, quality, information security
[37]	Introduction of a shared service center for telehealth	Service categories, effectiveness and efficiency evaluation
[38]	Addressing challenges related to diabetic retinopathy screening with telehealth	Screening adherence, patient outcomes

**Table 2:** Key elements/points emerging on the overview.

Study	Area of the Study	Key Points
[21]	Digital Health Interventions (DHIs)	- Digital health interventions (DHIs) provide innovative healthcare methods. - KPIs are crucial for evaluating healthcare quality. - Importance of stakeholder involvement in KPI development using methodologies like elicitation frameworks, factorial surveys, and Delphi studies. - Significant gap in evidence-based knowledge for DHIs. - Lack of standardized methodologies for KPI development. - Need for common methods to improve comparability and advance the field.
[22]	Telehealth Integration and Impact	- Teledermatology (TD) and conventional care pathways for non-melanoma skin cancer (NMSC) management, with time as a crucial KPI. - Unexpected finding: TD-managed patients were significantly older, but their total time spent in the process was not shorter compared to those managed conventionally.
[23]	Telehealth Integration and Impact	- Telehealth substitution rates at Princess Alexandra Hospital, focusing on telehealth-eligible and overall telehealth substitution rates. - Disparity between telehealth-eligible rate and overall telehealth substitution rate due to geographical constraints. - KPIs like telehealth activity as a reportable metric significantly influenced substitution rates.
[24]	Clinical Pharmacy Services	- Consensus on pharmaceutical care bundles and outcome measures for clinical pharmacy services. - Top-ranked KPI: proportion of patients where a pharmacist documents an accurate list of medicines during admission. - Consensus on sixteen outcome measures, including hospital-acquired complications and readmission due to medication misadventure. - Measurement of pharmaceutical care bundle delivery and its correlation with patient outcomes.
[25]	Chronic Disease Management	- Implementation of Fresenius Medical Care medical patient review CQI (MPR-CQI) and its impact on patients’ survival in end-stage kidney disease. - Significant reduction in mortality through improved KPI target achievement.
[26]	Telehealth Integration and Impact	- Evaluation criteria in telehealth, emphasizing KPIs for clinical outcomes, satisfaction, quality, and cost measures. - Variations in metric reporting among telehealth classes and study stages. - Frequent reporting of clinical outcomes and patient satisfaction, but rare reporting of other performance metrics.
[27]	Chronic Disease Management	- Quality of care comparison between telehealth and in-person visits using Health Care Effectiveness Data and Information Set (HEDIS) performance measures. - Telehealth exposure associated with better performance in chronic disease management and preventive care quality measures.
[28]	Telehealth Integration and Impact	- Factors associated with telehealth implementation: research performance, training, collaboration with the technology industry, and legislative frameworks. - Emphasis on consistent collection and routine analysis of patient outcomes after telehealth interventions. - Need for international legislation to support telehealth capacity building, reimbursement policy, and secure data sharing policies.
[29]	Technological Implementation in Healthcare	- Development and clinical evaluation of 5G usability test framework for preclinical diagnostics using mobile ultrasound and 5G network technology. - KPIs related to ultrasound image quality, transmission latency, and data throughput. - Positive ratings for the usability and clinical applicability of the ultrasound probe. - Successful bidirectional data transmission through the 5G network.
[30]	Evaluation Frameworks and Methodologies	- Face validity of the Model for Assessment of Telehealth (MAST) confirmed. - Suggestions for improvements in study design and data collection. - Emphasis on the need for larger studies to confirm results.
[31]	Emergency Medical Services (EMS)	- Longitudinal effects of tele-EMS system on EMS operations in Aachen. - Increased tele-EMS consultations and availability of onsite EMS physicians. - Improvements in operational efficiency, diagnostic patterns, and resource utilization through telehealth integration. - Reduction in teleconsultation duration and handling multiple cases simultaneously.
[32]	Telehealth Integration and Impact	- Integrated Telehealth Model (ITM) within the Centers for Medicare and Medicaid Services’ meaningful use (MU) program. - KPIs for incentivizing telehealth implementation and enhancing healthcare quality. - ITM Model presents a strategic approach to enhancing healthcare delivery through technology, clinical processes, and integrated care.
[33]	Telehealth Integration and Impact	- Potential of telehealth systems in managing chronic diseases and reducing NHS burden. - KPIs assessing cost-efficiency and healthcare outcomes. - Challenges in measuring impact on healthcare outcomes, such as reducing emergency room visits and unnecessary hospital visits.
[34]	Telehealth Integration and Impact	- Evaluation of telehealth services in hospitals, focusing on clinical, economic, and satisfaction perspectives. - Need for improved reporting of telehealth service implementation and planning strategies.
[35]	Technological Implementation in Healthcare	- Superior performance of novel video data transmission methods in 5G networks. - KPIs like collision error, propagation error, sensing error, and visual security with encryption. - Comparative analysis showing better performance of the proposed method over existing approaches.
[36]	Evaluation Frameworks and Methodologies	- Comprehensive evaluation framework for telehealth systems. - KPIs related to IT satisfaction, service provider and consumer satisfaction, cost, quality, and information security. - Need for further research to validate and enhance the framework.
[37]	Telehealth Integration and Impact	- “Shared service center” concept for supporting telehealth initiatives. - Emphasis on the significance of KPIs in evaluating effectiveness and efficiency. - Framework for assessing the impact and success of telehealth initiatives.
[38]	Chronic Disease Management	- Telehealth improving screening rates and reducing complications in diabetic retinopathy management. - KPIs guiding program evaluation and resource allocation. - New imaging platforms enhancing detection and grading. - Financial barriers persist in telehealth implementation.

**Table 3:** Connection of ISfTeH, ECHAlliance, DHS with the field of the KPIs.

Entity	Approach to KPIs
ISfTeH (International Society for Telemedicine and eHealth)	Provides indirect references to KPI-related themes through documents such as the “2018-19-INTERNATIONAL-TELEHEALTH-CODE-OF-PRACTICE.pdf”.
European Connected Health Alliance (ECHAlliance)	Emphasizes the procedural importance of KPIs, suggesting measures like outcome-based indicators. Documents highlight their relevance in heal-thcare settings.
Digital Health Society (DHS)	Acts as an enabler for multi-stakeholder dialogue on digital health policies, indirectly contributing to understanding and implementing KPIs in healthcare.

**Table 4:** Analysed Entity with the relevant focus on the KPI.

Entity	Key Points on KPIs
WHO (World Health Organization)	Emphasizes demonstrating benefits and addressing challenges in healthcare service provider transitions to telehealth. Proposes short, medium, and long-term evaluation KPis covering aspects like specialty expansion, teleconsultation volume, patient savings, and long-term patient monitoring.
Document by ATA	Highlights balancing clinical excellence with operational efficiency in telehealth. Focuses on patient experience and the integration of business systems. Identifies challenges including care quality measurement and patient data privacy concerns.
ACP	Advocates for integrating telehealth into existing quality measurement frameworks. Recommends adapting performance measures to telehealth environments while ensuring equity and quality standards are maintained.
NHS (National Health Service—UK)	Provides specific KPIs for Integrated Urgent Care (IUC), integrating telehealth into urgent care activities. Includes metrics such as call abandonment rates, response times, and the percentage of calls managed remotely or referred for further care.
Dubai Health Authority	Establishes guidelines for reporting telehealth KPIs, focusing on access and quality metrics. Examples include waiting times, referral percentages, population coverage, medication prescriptions, patient and staff satisfaction, and complaints handling.

## Data Availability

Not applicable.

## Supplementary Materials

The following supporting information can be downloaded at: https://www.mdpi.com/article/10.3390/healthcare12131319/s1, Box S1: Keys used in the Pubmed search.
