# Supplementary material for: Optimizing Telehealth: Leveraging Key Performance Indicators for Enhanced TeleHealth and Digital Healthcare Outcomes (Telemechron Study)
Source: Healthcare (Basel). 2024 Jul 1;12(13):1319. doi: 10.3390/healthcare12131319 (PMC11241174; doi:10.3390/healthcare12131319)
Supplement: Supplementary file 1 [file healthcare-12-01319-s001.zip › healthcare-3066873-supplementary.pdf]

*healthcare*

*Review*

# Optimizing Telehealth: Leveraging Key Performance Indicators for Enhanced TeleHealth and Digital Healthcare Outcomes (Telemechron Study)

**Sandra Morelli [1] , Carla Daniele[1], Giuseppe D'Avenio[1], Mauro Grigioni[1], and Daniele Giansanti [1,*]**

[1] Centro TISP,ISS,via regina Elena 299,00161, Roma
* Correspondence: daniele.giansanti@iss.it;

.

## Supplementary Materials

*telehealth[Title/Abstract]*
*telehealth[Title/Abstract] AND quality[Title/Abstract]*
*telehealth[Title/Abstract] AND quality[Title/Abstract] AND indicator[Title/Abstract]*

**Box S1.** Keys used in the Pubmed search.
